# Pharmacy Technicians’ Contribution to Counselling at Community Pharmacies in Denmark

**DOI:** 10.3390/pharmacy8010048

**Published:** 2020-03-23

**Authors:** Mira El-Souri, Rikke Nørgaard Hansen, Ann Moon Raagaard, Birthe Søndergaard, Charlotte Rossing

**Affiliations:** 1 Department of Research and Development, Pharmakon, Danish College of Pharmacy Practice, 3400 Hilleroed, Denmark; rnh@pharmakon.dk; 2 Danish Association of Pharmacy Technicians, 2500 Valby, Denmark; amr@farmakonom.dk; 3 Association of Danish Pharmacies, 1260 Copenhagen, Denmark; bis@apotekerforeningen.dk

**Keywords:** pharmacy technicians, counselling, drug-related problems, community pharmacy, OTC medication, prescription medication, non-medical products

## Abstract

(1) **Background:** pharmacy technicians are the largest group of staff at Danish community pharmacies and play a vital role in counselling customers on prescription medication, over-the-counter (OTC) medication and non-medical products. This is the first study carried out to specifically analyse how they contribute to counselling and identification of drug-related problems (DRPs) at Danish community pharmacies. (2) **Methods:** seventy-six pharmacy technicians from 38 community pharmacies registered data on all of their customer visits for five days, over a four-week period, between January and March 2019. Data were analysed in SPSS version 24. (3) **Results:** 58.9% of all registered customers (n = 10,417) received counselling. They identified DRPs for 15.8% of all registered customers (n = 2800). Counselling by pharmacy technicians solved, or partially solved, problems for 70.4% of customers with DRPs. Pharmacy technicians estimated that 25.2% of customers receiving counselling (n = 2621) were saved a visit to the general practitioner (GP). (4) **Conclusions:** as community pharmacists get more involved in complex services, it would be necessary to expand the roles of pharmacy technicians. Pharmacy technicians contribute to medication safety via counselling, and identifying and handling DRPs for all customers. This study documents the role of pharmacy technicians in customer counselling at Danish community pharmacies. It provides evidence to researchers and policy makers to support discussions on the future role of pharmacy technicians at community pharmacies.

## 1. Introduction

Pharmacy technicians (in Denmark called pharmaconomists), with a three-year degree, are the largest group of staff at Danish community pharmacies. Their roles are expansive and bear some resemblance to those of pharmacists in some countries. They play a vital role in counselling customers on prescription medication, over-the-counter (OTC) medication and non-medical products. In general, pharmacy technicians perform a broad array of tasks in community pharmacies, except for the more complex pharmacotherapeutic ones. In Denmark, pharmacy technicians can deliver services, such as Inhaler Technique Assessment Service, teaching and quality assurance. Some community pharmacy services, such as medication review and New Medicine Service, are restricted to be delivered by pharmacists. In Denmark, all pharmacy technicians are educated at the Danish College of Pharmacy Practice, Department of Education.

Denmark has a population of 5.7 million people; they are served by 237 community pharmacies, 254 branch pharmacies and two online pharmacies that offer prescription medication, OTC medication and a restricted selection of non-medical products [1]. This means that there is a community pharmacy or a branch pharmacy for every 11,600 inhabitants. Furthermore, there are 29 medicine distribution units that offer OTC medication, a restricted selection of non-medical products and pre-ordered prescription medication. Normally, counselling at these units is provided by pharmacy technicians [1]. Finally, there are 750 medicine distribution units, where only pre-ordered medicine is provided. No counselling is offered here as there are no pharmacists or pharmacy technicians, and unskilled staff are not allowed to give information on medicines [1]. Having all these different units ensures easy access to medicines in all regions of Denmark. 

Community pharmacies and the appurtenant branch pharmacies in Denmark provide medical supplies, information, counselling and preventive health services by pharmacy technicians, along with pharmacists, to ensure the safe use of drugs [2].

Drug-related problems (DRPs) are common and associated with economic and patient-related costs [3,4]. A drug-related problem is defined by Hepler and Strand as: “an event or a circumstance involving drug treatment that actually or potentially interferes with the patient’s experiencing an optimum outcome of medical care” [5]. Examples of DRPs are inappropriate drug use, adverse reactions and non-adherence. 

Previous research shows that pharmacists play a role in identifying DRPs for customers at community pharmacies and that they contribute to solving them [6,7,8,9,10,11,12,13]. Research on DRPs focuses on evaluating community pharmacist-driven programmes, mostly at community pharmacies, but also, for example, in care facilities for the elderly [10,11,14,15]. Previous and ongoing research also focus on evaluating counselling, including identification and solving of DRPs in daily practice in community pharmacies. Some of this research also included counselling provided by pharmacy technicians along with pharmacists [13,16,17]. 

Pharmacy technicians, in general, are being accorded greater scopes of practice in community pharmacies, and, in particular, Danish pharmacy technicians are playing a vital role in counselling customers [18,19,20]. A previous Danish study on DRPs in self-medication showed that pharmacists and pharmacy technicians identified DRPs for 21% of pharmacy customers presenting a symptom or requesting OTC medication [13]. However, it has, so far, not been documented how pharmacy technicians, in particular, contribute to counselling in general, and handling of DRPs at community pharmacies. It is crucial to document their contribution in order to feed the discussion of their future roles at community pharmacies.

The objective of this study is to map the pharmacy technicians’ counselling activities regarding prescription medication, OTC medication and non-medical products at community pharmacies in Denmark and to describe how pharmacy technicians identify and handle DRPs. 

This is the first study carried out to specifically analyse how pharmacy technicians contribute to counselling and DRP identification at Danish community pharmacies. The study was carried out in collaboration with the Danish Association of Pharmacy Technicians, the Association of Danish Pharmacies and Pharmakon–Danish College of Pharmacy Practice.

## 2. Materials and Methods 

The design of the study is quantitative, comprising a descriptive approach. 

All community pharmacies were invited to participate in the study through different media, including professional groups on LinkedIn and Facebook, newsletters from the Danish Association of Pharmacy Technicians, the Association of Danish Pharmacies and Pharmakon–Danish College of Pharmacy Practice. 

To ensure the representativity of community pharmacies in Denmark, a list of Danish community pharmacies was used, and every fourth community pharmacy on the list was called. The community pharmacies were selected so that the number of recruited community pharmacies in each region reflected the number of pharmacies as much as possible. Seventy-six pharmacy technicians from 38 community pharmacies (two pharmacy technicians from each pharmacy) from all Danish regions were recruited after responding to a nationwide invitation. Fourteen community pharmacies were recruited by phone by the research group.

**Figure 1:**
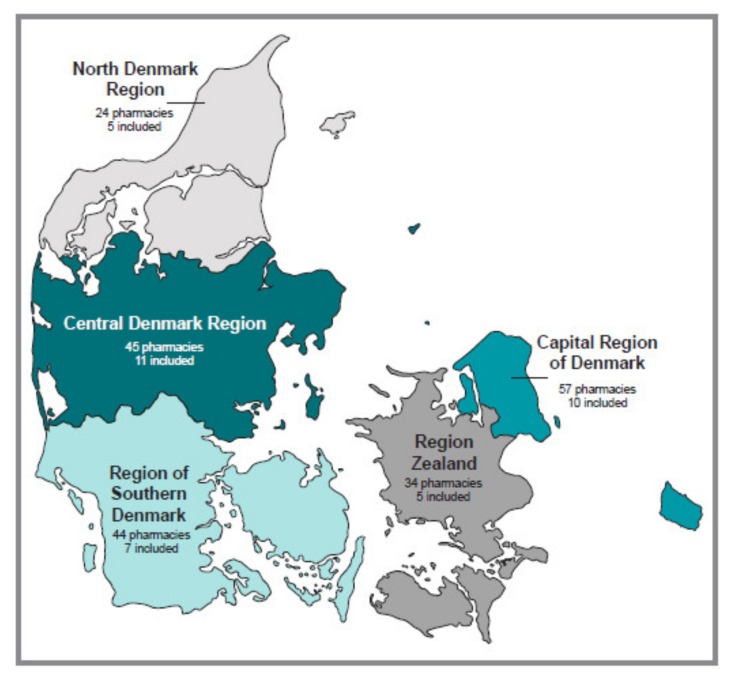
Number of community pharmacies included in each region.

The geographic distribution of the included pharmacies is illustrated in Figure 1.

The participating pharmacy technicians registered data on all their customer visits to the community pharmacies for five days over a four-week period between January and March 2019. They registered on different days of the week to cover the normal opening hours of most community pharmacies in Denmark.

At the beginning of the study period, the pharmacy technicians were introduced to the study through a webinar and self-study. They received training on registration consisting of an instruction and eight cases, which they were requested to solve before starting the registration process. During the study period, the research group held two question and answer (Q&A) webinars and could furthermore be contacted for support on registration. 

The registration was carried out in an electronic survey developed by researchers and representatives from the Danish Association of Pharmacy Technicians and the Association of Danish Pharmacies. It was also pilot-tested by two pharmacy technicians and adjusted afterwards prior to being used in the study. The registration had the following questions: gender and age of the customer, type of errand(s), type of identified DRP(s), counselling subject(s), solving of DRP(s) and whether the counselling saved a visit to the general practitioner (GP). Most of the questions could be answered by clicking on a drop-down menu to save time. All types of DRPs and the counselling subjects were defined by the research group and adjusted after the pilot study. All questions in the registration were answered by the pharmacy technicians and, therefore, the data is self-reported. 

In order to document the proportion of customers registered, the pharmacy technicians registered the total amount of customer visits received on the days chosen for registration. 

Data was analysed in SPSS version 24 (IBM Corp., Armonk, NY, USA). 

## 3. Results

**Figure 2:**
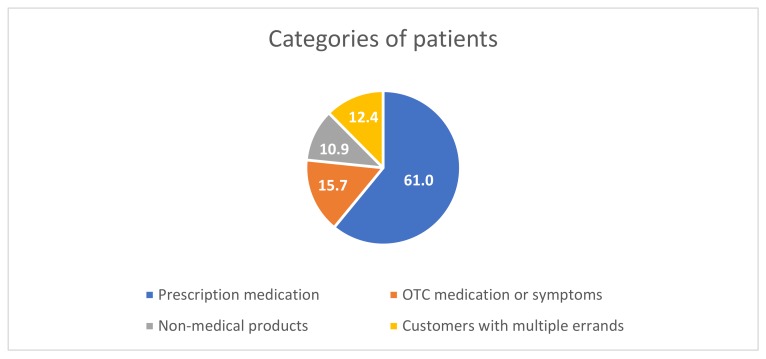
The categories of the customers in the study.

Information about a total of 17,692 customers was registered. Of these, 61.0% (n = 10,785) requested prescription medication, 15.7% (n = 2773) requested OTC medication or presented a symptom, 10.9% (n = 1937) requested non-medical products and 12.4% (n = 2197) inquired about a mix of the three categories (Figure 2). 

The results are presented for the total study population and for these subgroups:

- customers requesting prescription medication,
- customers requesting OTC medication or presenting a symptom,
- customers requesting non-medical products.

### 3.1. Description of the Study Population 

#### 3.1.1. Proportion of Customers Registered

According to the participating pharmacies check-out systems, they had a total of 21,621 customer visits on the days they registered. The total amount of customers registered was 17,692. That means that 82% of customer visits were registered.

#### 3.1.2. Gender and Age of the Total Study Population

Gender and age were registered for all 17,692 registered customer visits; 58.7% (n = 10,377) were women, 41.3% (n = 7315) were men. Moreover, 52.0% (n = 9199) were between 21 and 64 years old; 38.6% (n = 6835) were 65 years or older; 5.1% of the visits (n = 909) were about children (0–15 years). 

### 3.2. Counselling 

#### 3.2.1. Extent of Counselling in the Subgroups 

56.1% of customers requesting prescription medication (n = 6050) received counselling on one or more subjects. This was also the case for 68.0% of customers requesting OTC medication or presenting a symptom (n = 1885) and 46.7% of customers requesting non-medical products (n = 905). The reasons why customers did not receive counselling were: the drug/product was for someone else, the customer was familiar with the drug/product, the customer had received counselling at the general practitioner (GP)/hospital, the customer had a language barrier or the customer was a doctor/nurse. 

#### 3.2.2. Counselling Subjects 

Table 1 presents an overview of the subjects covered by the counselling.

“Drug/product use” was the most frequent subject in counselling for the total study population and in all subgroups. It accounted for 58.4% of all customers who received counselling; 56.4% of customers who requested prescription medication received counselling; 48.8% of customers who requested OTC medication or who presented a symptom received counselling and 67.6% of customers who requested non-medical products received counselling.

In counselling of customers who requested prescription medication, the second and third most frequent subjects were “adverse reactions” and “effect of the drug” (30.9% and 29.0% of customers received counselling). 

In counselling costumers who requested OTC medication/presenting a symptom, the second and third most frequent subjects were “effect of the drug” and “personal care” (32.9% and 24.6% of customers received counselling). 

In counselling costumers who requested non-medical products, the second and third most frequent subjects were “personal care” and “effect of the drug” (34.0% and 27.6% of customers received counselling). 

The pharmacy technicians estimated that their counselling saved 25.2% of the customers (n = 2621) a visit to the GP, broken down by 22.9% of customers requesting prescription medication (n = 1383), 30.7% of customers requesting OTC medication or presenting a symptom (n = 578) and 17.7% of customers requesting non-medical products (n = 160).

### 3.3. Occurrence and Types of DRPs

One or more DRPs were identified for 15.8% of the total study population (n = 2800), 17.8% of customers requesting only prescription medication (n = 1917), 12.7% of customers requesting only OTC medication or presenting a symptom (n = 352), and 6.9% of customers requesting only non-medical products (n = 133). 

Table 2 shows the types of DRPs identified in the study. The types of DRPs are divided into two categories: (1) treatment effectiveness and safety problems and (2) logistical problems. 

#### 3.3.1. Types of DRP—Treatment Effectiveness and Safety Problems

The most frequent DRPs related to treatment effectiveness and safety vary between the subgroups (Table 2). “Adverse reactions” and “non-adherence” were the most common DRPs for customers requesting prescription medication (8.1% and 8.0% of customers with DRPs). 

The most common DRPs relating to OTC medication or a symptom were “inappropriate drug/product”, “symptom that requires a visit to the GP” (17.6% and 17.3% of customers with DRPs). 

Regarding customers requesting non-medical products, the most frequent DRPs were “inappropriate drug/product” and “symptom that requires a visit to the GP” (27.1% and 8.3% of customers with DRPs). 

#### 3.3.2. Types of DRP—Logistical Problems

The most frequent logistical problems identified for customers requesting prescription medication were that the prescription was not available, the drug/product requested was not available and the prescription was incomplete or inaccurate (30.6%, 22.7% and 8.7% of customers with DRPs).

A frequent DRP for customers requesting OTC medication, or presenting a symptom, was that the drug/product they requested was not available (12.5% of customers with DRPs). This was also a frequent DRP for customers requesting non-medical products (21.1% of customers with DRPs).

#### 3.3.3. Solving of Identified DRPs

The pharmacy technicians estimated that 51.9% of customers with DRPs got their DRP solved, 18.5% of customers with DRPs got their problems partially solved and 20.5% of customers with DRPs did not get their problems solved (n = 1452; n = 517; n = 575). For 9.1% of customers with DRPs, the pharmacy technicians answered “don’t know” or gave no answer (n = 256).

## 4. Discussion

### 4.1. Discussion of the Results 

#### 4.1.1. Proportion of Customers Receiving Counselling

A study mapping Norwegian community pharmacy counselling shows that 60% of the customers receive counselling [17]. Although 80% of the participants in the Norwegian study are community pharmacists, and all of the participants in this study are pharmacy technicians, the proportion of customers receiving counselling in the two studies is comparable.

#### 4.1.2. Counselling Customers Requesting OTC Medication or Presenting a Symptom

In an earlier Danish study mapping DRPs in OTC medication customers, identified by pharmacy technicians and community pharmacists, the most frequent counselling subjects were “counselling on self-medication”, “personal care” and “recommendation of a drug/product” [13]. There are similarities between the findings in the two Danish studies because “counselling on self-medication” can cover both of the following subjects: “drug/product use” and “effect of the drug”, which were the two most frequent counselling subjects for customers requesting OTC medication or presenting a symptom in this study. In a German study mapping DRPs in OTC medication customers, identified by community pharmacists, the most frequent subjects were “referral to a physician” and “switching to a more appropriate drug” [21]. Pharmacy technicians in this study referred customers to their GP, and they recommended more appropriate drugs/products to some of the customers. However, these two categories were not the most frequent ones. These results show that pharmacy technician counselling is comparable to counselling delivered by community pharmacists, especially in a Danish setting. 

#### 4.1.3. Types of DRPs in the Total Study Population

In a German study mapping the DRPs encountered in community pharmacies, identified by pharmacists, the most frequent types of DRPs were “evidence of drug-drug interaction in the literature”, “incomplete or unreadable prescription” and “drug not on the market” [7]. It is remarkable that drug-drug interaction is the most frequent DRP in the German study. There are two evidence-based electronic databases in Denmark in which health professionals and patients, respectively, can check for drug-drug interactions. It must be checked if the interactions are clinically relevant; most often only a few of them are. Drug-drug interactions are identified in this study, but are not among the most frequently registered DRPs; but of course, it can be crucial when it is clinically relevant. Otherwise, the next two frequently registered types of DRPs in the German study are similar to the two most frequently registered types of DRPs in this study. Thus, the types of DRPs identified by pharmacy technicians are comparable to those found by pharmacists.

Drug shortage is an international problem and a known and increasing problem in Danish community pharmacies [22,23]. The Association of Danish Pharmacies was contacted by the research group, and they had not detected any extraordinary fluctuation in the frequency of drug shortages in the study period.

A high rate of unavailable prescriptions was shown in this study. A newly published study shows that unavailable prescriptions occur in 1% of all dispensing in Danish community pharmacies. Miscommunication between the patient and GP seems to be the primary source of unavailable prescriptions [24].

#### 4.1.4. Occurrence of DRPs for OTC Medication Customers

DRPs were identified for 15.8% of the total study population and for 12.7% of customers requesting OTC medication or presenting a symptom. The earlier German study documented DRPs in 17.6% of all cases [21]. The earlier Danish study documented DRPs in 21.0% of OTC medication customers [13]. Both studies showed a higher occurrence of identified DRPs in OTC medication customers than this study. It is important to mention that the percentages in the Danish studies are calculated as percentage (%) per customer. The percentages in the German study are calculated as percentage (%) per case or request. So, the Danish results are not comparable with the results from the German study. The percentage in the German study may be lower than 17.6%, as it can be assumed that some of the customers had multiple DRPs. The high occurrence of DRPs identified in the earlier Danish study may be due to extra focus on OTC medication customers during the study period, as it was the aim of the study. 

#### 4.1.5. Types of DRPs in Customers Requesting OTC Medication or Presenting a Symptom 

The most frequent types of DRPs identified for customers requesting OTC medication or presenting a symptom were “inappropriate drug/product”, “symptom that requires a visit to the GP”, “adverse reaction” and “duration of treatment too long” (17.6%, 17.3%, 9.4% and 8.8% of customers with DRPs). There are similarities to the German study, which reported that the most frequent DRPs were “self-medication inappropriate”, requested drug inappropriate” and “intended duration of drug use too high”. In the earlier Danish study, the most frequent types of DRPs were: “the choice of medication is not appropriate/optimal for the condition” (44.8%), “too little of the drug is being taken” (17.0%), “the drug is taken for too long” (15.0%) and “adverse reactions”(13.8%) [13]. The types of identified DRPs are also similar in the two Danish studies. It is remarkable that pharmacy technicians in this study identify symptoms that require a visit to the GP, which probably leads to early diagnosis and treatment, and in this manner, promotes patient safety. 

#### 4.1.6. Pharmacy Technicians’ Contribution to Counselling and Handling of DRPs

This study documents that pharmacy technicians contribute to counselling in Danish community pharmacies. The subgroup with the highest proportion of customers receiving counselling represents those who request OTC medication or present a symptom (68.0% compared to 58.9% in the total study population). This is probably because most of these customers have not received any prior counselling from a healthcare professional on their OTC medication or their symptom, so they seek counselling at the community pharmacy, and this counselling could be very important for these customers.

Pharmacy technicians have shown that they can identify DRPs for all subgroups. The prevalence of DRPs identified by pharmacy technicians for customers requesting only prescription medication is comparable with the prevalence of DRPs identified by community pharmacists. A Belgian study on the identification and handling of DRPs by community pharmacists in the dispensing process documents that at least one DRP is found in 9869 on a total of 64,962 prescriptions (15%) [25]. The pharmacy technicians in this study solved, or partially solved, DRPs for 70.4% of customers with DRPs. This is also comparable with the Belgian study, where the community pharmacists solved almost 75% of the identified DRPs [25]. Pharmacy technicians contribute to patient safety. In particular, this is documented by the high extent of counselling provided to customers requesting OTC medication or presenting a symptom, and identification and solving of DRPs for customers requesting non-medical products. These customers would probably not have been counselled on DRPs if they had chosen to buy their products from other outlets than the community pharmacy. 

### 4.2. Method Discussion 

In order to ensure representativity, a couple of initiatives were carried out. 

First, the community pharmacies were selected to reflect the number of pharmacies in each region. 

Second, the participants were instructed to choose five days over a four-week period to register all visits and to choose different days of the week to avoid bias. The collected data do not indicate on which days of the week the registration took place, and we therefore cannot tell if the participants followed this instruction. If we look at the proportion of customers registered, which was 82% on average, it can be assumed that the proportion is high enough to conclude that the collected data is representative.

Third, the participants received training on the registration consisting of an instruction and eight cases, which they were requested to solve before starting the registration process. The participants had access to the correct answers, but it might have been better if they had received feedback on their answers from the researchers before starting their registration. 

Due to the study design, the pharmacy technicians collected data on their own counselling activities—self-reported data. Questions, such as whether the counselling saved visits to the GP, were answered by the pharmacy technicians by self-estimation. They were asked to estimate whether the customer would have contacted the GP if they had not received counselling from the community pharmacy, and then, whether the counselling had saved the customer a visit to the GP. There might be a bias here. The wording of the question makes it difficult to give an answer as the pharmacy technicians are asked to estimate two scenarios at the same time. 

## 5. Conclusions

Pharmacy technicians contribute to medication safety by counselling and identification and handling of DRPs for customers requesting prescription medication, OTC medication (including those presenting a symptom) and non-medical products.

Moreover, 58.9% of all registered customers (n = 10,417) received counselling. 

Pharmacy technicians identified DRPs for 15.8% of all registered customers (n = 2800). Counselling by the pharmacy technicians solved, or partially solved, problems for 70.4% of customers with DRPs. The pharmacy technicians estimated that 25.2% of customers receiving counselling (n = 2621) were saved a visit to the GP.

As community pharmacists get more involved in complex services, it would be necessary to expand the roles of pharmacy technicians. This study maps and documents the important role of pharmacy technicians in counselling at Danish community pharmacies. It provides evidence to researchers and policy makers to support the discussion of the future role of pharmacy technicians in community pharmacies. 

## Figures and Tables

**Table 1:** Subjects covered in counselling for the total study population and the three subgroups. The most frequent counselling subjects are highlighted * (threshold value 15.0%).

Subject	All Customers Who Received Counselling (N = 10,417) % (n)	Customers Requesting Prescription Medication Who Received Counselling (N = 6050) % (n)	Customers Requesting OTC Medication or Presenting a Symptom Who Received Counselling (N = 2773) % (n)	Customers Requesting Only Non-Medical Products Who Received Counselling (N = 905) % (n)
Personal care	28.7% (2987) *	23.4% (1414) *	24.6% (683) *	34.0% (308) *
Adverse reactions	26.3% (2743) *	30.9% (1870) *	15.1% (419) *	3.6% (33)
Effect of the drug/product	34.6% (3604) *	29.0% (1754) *	32.9% (913) *	27.6% (250) *
Interactions	4.3% (448)	3.5% (214)	4.5% (126)	2.2% (20)
Drug/product use	58.4% (6079) *	56.4% (3410) *	48.8% (1352) *	67.6% (612) *
Adherence	17.6% (1830) *	19.2% (1162) *	9.2% (256)	7.8% (71)
Recommendation for a drug/product	7.4% (771)	3.6% (218)	9.2% (256)	12.9% (117)
Recommendation for another drug/product than the one requested	4.5% (467)	1.7% (104)	5.9% (164)	8.4% (76)
Recommendation for automated dose dispensing	0.1% (14)	0.2% (13)	0% (0)	0% (0)
Drug/product substitution	18.2% (1901)	25.2% (1525) *	3.5% (96)	1.8% (16)
Reimbursement	6.5% (680)	9.0% (542)	1.3% (36)	0.9% (8)
Medication waste	0.7% (69)	0.9% (57)	0.3% (7)	0.1% (1)
The pharmacy technician contacted the GP	1.2% (121)	1.7% (103)	0.1% (4)	0.1% (1)
The pharmacy technician recommended the customer to contact a GP	12.8% (1336)	13.9% (841)	8.4% (233)	5.6% (51)
Total	221.3% (23,050) **	218.6% (13,227) **	163.9% (4545) **	172.8% (1564) **

** The percentage is over 100% due to the fact that some customers received counselling on more than one subject. GP = general practitioner; OTC = over-the-counter.

**Table 2:** Drug-related problems (DRPs) in the total study population and in the three categories of customers. The most frequent DRPs are highlighted * (threshold value 8.0%).

DRP	All Customers with DRPs, (N = 2800) % (n)	Customers with DRPs Requesting Only Prescription Medication, (N = 1917) % (n)	Customers with DRPs Requesting Only OTC Medication or Presenting a Symptom, (N = 352) % (n)	Customers with DRPs Requesting Only Non-Medical Products, (N = 133) % (n)
Treatment effectiveness and treatment safety problems				
Inappropriate drug/product	5.8% (163)	1.5% (28)	17.6% (62) *	27.1% (36) *
Contraindication	1.1% (30)	0.4% (7)	3.1% (11)	1.5% (2)
Double dose	2.2% (62)	1.4% (27)	4.5% (16)	1.5% (2)
Interaction	1.8% (51)	1.0% (19)	3.4% (12)	2.3% (3)
Drug dose too high	2.9% (81)	2.3% (45)	5.1% (18)	2.3% (3)
Drug dose too low	3.4% (96)	2.3% (44)	7.4% (26)	5.3% (7)
Duration of treatment too long	2.9% (81)	1.4% (26)	8.8% (31) *	0.8% (1)
Duration of treatment too short	1.8% (50)	1.3% (25)	3.7% (13)	0.8% (1)
Adverse reaction	9.2% (257) *	8.1% (156) *	9.4% (33) *	6.0% (8)
Symptom that requires a visit to a GP	6.4% (179)	3.3% (64)	17.3% (61) *	8.3% (11) *
Problem with practical use of drug/product	4.1% (116)	3.9% (75)	5.7% (20)	6.0% (8)
Non-adherence	7.6% (214)	8.0% (153) *	5.1% (18)	5.3% (7)
Logistical problems				
Prescription is incomplete or inaccurate	6.4% (179)	8.7% (166) *	-	-
Product or prescribed drug not available	20.6% (578) *	22.7% (436) *	12.5% (44) *	21.1% (28) *
Prescription not available	24.0% (673) *	30.6% (586) *	-	-
Other problems				
Other problems	9.5% (265) *	3.1% (60)	-	12.0% (16)
Total	104.0% (3075) **	100.0% (1917)	103.6% (365) **	100.0% (133)

** The percentage is over 100% due to the fact that some customers had more than one DRP.
